# Prescribing Physical Activity for the Prevention and Treatment of Osteoporosis in Older Adults

**DOI:** 10.3390/healthcare5040085

**Published:** 2017-11-06

**Authors:** Lachlan B. McMillan, Ayse Zengin, Peter R. Ebeling, David Scott

**Affiliations:** 1 School of Clinical Sciences at Monash Health, Monash Medical Centre, Monash University, Clayton, VIC 3168, Australia; ayse.zengin@monash.edu (A.Z.); peter.ebeling@monash.edu (P.R.E.); david.scott@monash.edu (D.S.); 2 Department of Medicine, Melbourne Medical School (Western Campus), The University of Melbourne, St Albans, Melbourne, VIC 3021, Australia; 3 Australian Institute for Musculoskeletal Science (AIMSS), Sunshine Hospital, St Albans, Melbourne, VIC 3021, Australia

**Keywords:** physical activity, exercise, bone mineral density, osteoporosis, resistance training, weight-bearing

## Abstract

Osteoporosis is an age-related disease, characterised by low bone mineral density (BMD) and compromised bone geometry and microarchitecture, leading to reduced bone strength. Physical activity (PA) has potential as a therapy for osteoporosis, yet different modalities of PA have varying influences on bone health. This review explores current evidence for the benefits of PA, and targeted exercise regimes for the prevention and treatment of osteoporosis in older adults. In particular, the outcomes of interventions involving resistance training, low- and high-impact weight bearing activities, and whole-body vibration therapy are discussed. Finally, we present recommendations for future research that may maximise the potential of exercise in primary and secondary prevention of osteoporosis in the ageing population.

## 1. Introduction

Physical activity (PA) and exercise have long been recognised as cornerstones of chronic disease prevention and management, due to their beneficial effects on clinical endpoints in a range of diseases, including many associated with ageing [1]. Greater levels of PA have repeatedly demonstrated an association with better health and quality of life [2,3], whilst, conversely, low PA is associated with negative health outcomes, including obesity, type 2 diabetes mellitus [4], and mortality [5]. A wide range of deleterious associations have also been observed with physical inactivity, or sedentary behaviour [6,7,8], which has been described as the major public health problem of our time [9]. Musculoskeletal health appears to be particularly compromised by both sedentary behaviour and/or low levels of PA [10,11]. Equally, the musculoskeletal system appears to function optimally with at least moderate quantities of PA and exercise [12,13,14], where PA is defined as incidental daily activities such as walking for transport, work or home duty activities, and exercise representing a form of activity, undertaken with a strict purpose to meet a specific target [15]. 

Osteoporosis is characterised by low bone mineral density (BMD), and poor bone geometry and microarchitecture, which confer an increased risk of minimal-trauma fractures associated with significant morbidity [16]. Hip fractures alone are associated with 20% mortality within 12 months, with higher rates in urbanised countries [17,18]. Importantly, minimal trauma fractures place an enormous burden on the economy and health care system. In Australia it is estimated that hip fractures cost an average of $23,000, and potentially over $33,000 in health care expenditure [19]. 

Experts have recently highlighted “a crisis in the treatment of osteoporosis” [20] as both prescription and adherence to pharmacotherapy regimes have decreased in recent years, which may be due to increased awareness of severe, yet rare, side effects of first-line treatment drugs; bisphosphonates. Whilst education for both clinicians and patients is likely key to improving prescription and compliance rates for osteoporosis medications, this highlights the importance of identifying non-pharmacological therapies for osteoporosis prevention and treatment. 

This narrative review will discuss the role that PA and exercise play in osteoporosis prevention and management, and how different exercise modalities contribute to bone health in older adults.

## 2. Mechanism of PA and Exercise Effects on Bone Health

**Figure 1:**
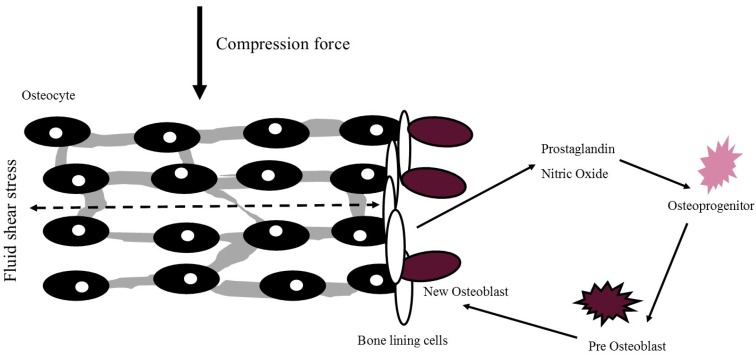
Cellular mechanism of mechanotransduction with new osteoblast formation. Adapted from Turner & Pavalko (1998).

Whilst the exact mechanism of osteogenesis via PA and exercise is yet to be fully elucidated due to challenges in studying cellular bone responses in vivo, it is likely that activity induces an anabolic or homeostatic effect on bone via mechanotransduction. These mechanisms have been particularly well described by Turner and Duncan and are summarised in Figure 1 [21,22]. Briefly, fluid movement within the extracellular matrix of bone exerts force on osteocytes and bone lining cells. This subsequently triggers the release of nitric oxide and prostaglandin, which lead to division and differentiation of osteoprogenitor cells. Pre-osteoblasts consequently mature to osteoblast cells and affix to the surface of the matrix to begin the production of new bone.

Muscular contractions may also induce this extracellular fluid shear stress within the bone matrix, producing deformations in bone [22]. Similarly, gravitational impacts produce deformations via fluid shear stresses and subsequent mechanotransduction [21,22,23,24,25]. However, these may have limited effects on organism-wide BMD as the skeletal sites most proximal to the engaged muscle groups or sites of gravitational impact during training are likely to experience the greatest increases in BMD [26], while those not engaged may see little adaptation. This notion is supported by cross-sectional studies that have demonstrated greater radial BMD in the dominant versus non-dominant arm of tennis players [27], and greater leg BMD in sprinters [11]. Nevertheless, few studies have investigated site-specific responses to exercise interventions [28], with primary study outcomes in this field generally focused on clinically relevant sites for osteoporosis, such as the total hip, femoral neck, or lumbar spine.

## 3. Modalities of PA and Exercise

### 3.1. Progressive Resistance Training

Progressive resistance training (PRT) is a common form of recreational exercise, generally involving weight-lifting and/or resistance bands and cables. Such training aims to develop muscle hypertrophy and strength through a range of isolated exercises. PRT is perhaps the most widely researched exercise modality targeting preservation of BMD in older adults [26,29,30,31,32,33,34,35,36,37,38].

Cross-sectional studies have demonstrated that weight lifters and strength-trained athletes have higher BMD than control or other athletic populations [39]. Karlsson et al. reported that adult weight lifters (mean ± SD age 33 ± 11 years) had 13% higher lumbar spine BMD compared with controls [40], and professional tennis players had 19% greater ulna diameter in their stroke compared to the contralateral arm [27]. However, as it is not possible to derive a causal relationship from these studies, research investigating the efficacy of such forms of exercise has largely focused on interventional trials.

An important goal for exercise trials is to assess efficacy in populations most at risk of fracture, yet limited studies have focused on older adults with, or at risk of developing, osteoporosis, with many excluding such individuals due to perceived safety concerns. However, an ongoing Australian study focused on post-menopausal women with low BMD (lumbar spine T-score < −1.0) has shown significant improvement in femoral neck (+2.8%) and lumbar spine BMD (+3.3%) during an eight-month supervised PRT intervention compared to an unsupervised home-based intervention [29]. These preliminary results highlight the effectiveness of PRT in older adults with poor bone health. Importantly, the study has reported no adverse events and a high exercise compliance rate of 87%. 

Supporting these results, Nelson et al. demonstrated in 40 post-menopausal Caucasian women between the ages of 50–70 years, a 12-month intervention of high-intensity resistance strength training increased femoral neck and lumbar spine BMD by 0.9% and 1.0%, respectively [41]. A similar intervention by Huovinen et al. also demonstrated that a 16-week PRT intervention, involving exercises such as leg presses, abdominal crunches, and other large muscle group exercises improved total hip BMD by 6% [42]. The wide variance in BMD improvements reported between these studies may be attributable to differences in exercise protocols, study populations, or methods of BMD assessment. Indeed, Huovinen et al. recruited post-menopausal women with low hand grip strength, as opposed to healthy post-menopausal women targeted by Nelson and colleagues. It stands to reason that post-menopausal women with low strength are able to produce more significant gains in strength given their initial deficit, and this in turn may correspond to larger adaptations within bone. However, it should also be noted that Huovinen et al. measured hip BMD by computed tomography rather than dual energy X-ray absorptiometry (DXA). Computed tomography, unlike DXA, relies on multiple X-ray scans at various angles, compiled into a two-dimensional image, with output data converted from Hounsfield units to BMD (g/cm^2^) through calibration phantoms [42]. Conversely, DXA directly assesses BMD via two X-ray beams of varying energy, with the differences in absorption of each ray corresponding to the density of the structure. These subtle differences may in part explain differences in BMD changes between these studies.

Fewer PRT-based interventions have been trialled in men exclusively [36,43,44], with two studies failing to show meaningful significant increases in BMD compared with control. A 12-month randomised control trial involving 143 men between the ages of 55–80 years compared the effect of three weekly supervised PRT sessions versus an active control who were instructed to walk for 30 min three times a week. Interestingly, both the PRT and active control group demonstrated significant positive changes in BMD at the total hip and trochanter of 0.9%, yet there was no difference between the groups [36]. Similarly, Ryan et al. did not show significant differences between intervention and inactive control groups within a 16-week intervention of upper and lower body PRT. Furthermore, neither group demonstrated significant improvement in lumbar spine or whole body BMD from the baseline [45].

Conversely, Kukulijan et al. conducted a 12-month trial involving 180 men aged 50–79 years, and demonstrated that their PRT exercise program was able to significantly improve lumbar spine BMD by 1.5% and total hip by 0.7% compared with control, although the addition of supplementation with vitamin D-fortified milk to this protocol may have influenced BMD changes [46]. 

Despite these conflicting results regarding improvements in BMD, PRT in men appears to be effective at least in maintaining BMD over time [36,43]. The aetiology of varying BMD response between men and women is likely multifactorial, attributable to body compositional, hormonal, or physiological differences amongst others.

The *Osteo*-*cise* program by Gianoudis et al. represents a larger scale PRT intervention to improve BMD performed in 162 community-dwelling men and women, involving three sessions per week for a period of 12 months. The program also involved a behavioural change program, designed to increase adoption and adherence, and improve awareness of osteoporosis. The PRT program demonstrated a significant 1.1% improvement in BMD at the lumbar spine, and 1.0% at the femoral neck, compared with control participants [32]. It should be noted that this study included both high-velocity PRT and impact weight-bearing exercise (which will be discussed later in this review), and as such it is unclear whether BMD improvements occurred as a result of PRT, impact exercise, or a combination of both. Moreover, Gianoudis et al. reported modest compliance of 59% to the exercise intervention with 40% of trained participants reporting musculoskeletal complaints, albeit most were regarded as minor. It is important to develop strategies to identify potential participants who are most likely to obtain improvements in BMD, whilst also lowering risk for adverse events and poor compliance in order to maximise outcomes of bone-targeted exercise interventions. 

Discrepancies in exercise protocols are likely to be a factor contributing to heterogeneity of results for PRT on BMD. Whilst Exercise and Sports Science Australia (ESSA) has released a position statement regarding exercise in older adults for prevention of osteoporosis [47], no consensus PRT protocol has been developed and validated in relation to positive change in BMD. As a result, study exercise protocols often vary markedly in regards to sites targeted, modes of delivery (e.g., group or individual; at home or a gym), methods of exercise progression, frequency, intensity and numbers of sets and repetitions. Moreover studies often involve exercise delivered in multi-modal format [32,46,48], and thus the effect of PRT alone is more difficult to singularly elucidate as previously mentioned.

On the whole, however, PRT appears to be an effective modality of increasing BMD in older adults, yet perhaps most effectively in women. PRT may be most effective in older adults with functional deficits or those most at risk of osteoporosis development. Future studies should aim to develop consistent protocols to determine independent effects of PRT on BMD in older adults, including men [49].

### 3.2. Ambulatory Activity

During the course of ageing, total PA declines progressively, with walking predominating as the most common form of PA in older adults [50,51,52]; whilst daily walking activity is associated with a range of positive health outcomes, its potential for increasing or maintaining BMD during ageing is less convincing [53,54]. Walking at usual pace likely has minimal benefit for muscle hypertrophy and generating gravitational impact, and so large amounts of walking are likely necessary to confer significant BMD improvement. Indeed, walking for at least 4 hours a day has been associated with a 41% reduction in hip fracture risk in post-menopausal women [55]; however, this may be explained by reductions in fall risk rather than improvements in BMD, and notwithstanding is an unrealistic target for the majority of older adults.

A number of different methods are available to quantify the amount of walking and ambulatory activity performed by older adults. The Healthy Ageing Initiative (HAI) recently reported that self-reported time spent walking is not associated with lower-limb BMD parameters [14]. However, self-reporting instruments may overestimate activity levels [56] and as a result objective methods such as pedometers or accelerometers are recommended for assessment of free-living PA. 

A cross-sectional analysis of the Tasmanian Older Adults Cohort study (TASOAC) including 875 older adults (51% women) who used pedometers for seven days demonstrated that pedometer-determined steps per day was positively associated with total hip BMD, but only in individuals over the age of 65 years [57]. For 65-year-old men and women, increasing steps per day by 25% was associated with up to 1.8% and 1.4% higher hip BMD respectively. It is not possible to determine causality from this cross-sectional study; the observed associations are perhaps a function of forms of activity other than walking (e.g., running or jumping) that may be more osteogenic. Pedometers provide information only on the number of steps completed during daily ambulatory activities, but are unable to quantify the intensity associated with these activities.

As such, accelerometers have been deployed in order to provide an objective measure of daily PA intensity. Wu et al. examined associations between seven-day accelerometer-derived PA and BMD in a cohort of 309 women (50 ± 5 years). Total PA was positively associated with femoral neck BMD (β, 95% CI; 0.011 g/cm^2^, 0.003 to 0.019), while moderate and vigorous intensity PA (MVPA) was also positively associated with femoral neck BMD (0.005 g/cm^2^, 0.0007 to 0.0094). Interestingly, light-intensity PA was not associated with any bone health outcomes. Indeed, captured MVPA may not be purely ambulatory, yet the results reaffirm initial findings indicating that walking alone, often regarded as light intensity, may have little benefit for osteogenesis.

Interventional trials have been conducted to overcome the inherent limitations of inferring causality on relationships between ambulatory activity and BMD from cross-sectional studies. As with other exercise modalities, these interventional studies have been limited by significant heterogeneity in protocols, particularly the intensity of walking activity prescribed. Studies have examined the effects of walking interventions at moderate ‘brisk’ pace [58,59,60,61] and Brooke-Wavell et al. have demonstrated in multiple trials that brisk walking may provide limited improvement in BMD, but may be effective in maintenance of BMD [59,61]. Specifically, in a group of 38 post-menopausal women, walking produced a mean improvement of +0.001 g/cm^2^ BMD at the calcaneus, compared to a concomitant decrease of −0.010 g/cm^2^ (*p* = 0.04) in the control group [59].

A meta-analysis of walking-only interventions in men and women over the age of 50 years, including 10 studies of BMD measurements at the lumbar spine, femur, and calcaneus, demonstrated a significant positive effect on lumbar spine BMD only, but with weak effect size [62]. A recent Cochrane review also examined the efficacy of walking exercise as a treatment for post-menopausal osteoporosis, including three trials [60,63,64] with a total of 156 participants. Meta-analysis of these studies demonstrated that walking had a significant, positive effect on BMD at the lumbar spine and total hip [65], yet also failed to demonstrate a meaningful effect size. This lack of a meaningful benefit of walking for BMD has been highlighted by the ACSM position stand on exercise for older adults [54].

Ambulatory activity presents an attractive option as an osteoporosis therapy, given the associated benefits to overall health status and physical function, and high feasibility in older adult populations. As studies to date have demonstrated heterogeneity in quantification of ambulatory activity, walking intensity, and intervention duration, along with small sample sizes, it is difficult to make a conclusive assessment regarding the effects of walking interventions on BMD in older adults. However, current evidence suggests walking does not generate gains in BMD, but may nevertheless maintain bone homeostasis during ageing, particularly when performed at higher intensities.

### 3.3. High-Impact Activity

High-impact activity may be the most widely utilised form of exercise to reduce the burden of osteoporosis in older adults [28,66,67,68,69,70,71], perhaps owing to the rationale that gravitational impact may be particularly osteogenic. Consisting of brief, high-impact exercises such as hopping, skipping, or jumping, such activity may influence several factors important in preventing fractures, including increases in BMD and decreases in fall risk via improvements in balance and proprioception along with gains in muscle strength and power [32,72,73]. 

Cross-sectional research has been limited in this space, as quantification of impact loading during free-living PA is difficult. The Bone Specific Activity Questionnaire (BPAQ) attempts to address this gap, with a set of specific questions developed to gain a comprehensive understanding of lifetime PA and its potential osteogenic influence. BPAQ utilises novel algorithms along with measured ground reaction forces (GRFs) to make inferences regarding habitual impact PA [74]. In a cohort of 36 healthy middle-aged and older men, BPAQ scores were significantly positively correlated with femoral neck (*r* = 0.42) and total hip (*r* = 0.36) BMD [75]. However, as previously discussed, self-reported questionnaires are prone to recall bias, and recent research has therefore focused on objective measures of high-impact PA.

Several seminal studies by Hannam and Deere have described the algorithmic classification of high-impact activity from hip-worn accelerometers [70,76,77]. It was demonstrated that accelerometer-derived impacts were low in older adults, with limited impacts at >1.5 g, well below the 4 g believed to be requisite for stimulating osteogenesis in younger adults [70,78,79]. 

Using an accelerometer to determine free-living PA in a cohort of 408 women (median age, 77 years), Hannam et al. demonstrated that impacts of >1.5 g were not related to BMD at the lumbar spine, total hip, or femoral neck. These high impacts were, however, related to other indices of bone health, particularly bone geometry characteristics such as tibial periosteal circumference, cross-sectional moment of inertia, and stress-strain index, assessed by computed tomography. Importantly, it was also demonstrated that whilst high impact may not be positively associated with BMD, low impacts were inversely associated with hip (β; *p*, −0.071; 0.02) and lumbar spine BMD (−0.161; <0.01). Moderate impacts were also inversely associated with BMD at the lumbar spine [13]. This study highlights that whilst free-living high-impact PA may not be sufficient to generate increases in BMD, it may like other forms of PA be an important factor in the maintenance of BMD during ageing.

A number of randomised control interventional studies investigating the effect of high-impact exercise programs have been conducted in older adults [67,71,80,81] and have shown varying results, briefly summarised in Table 1. 

A randomised control trial investigating the efficacy of high-impact exercise on BMD demonstrated significant improvement in BMD at the femoral neck when compared to the baseline and control. This study engaged participants in an hour-long class, 2–3 times per week. The high-impact activity consisted of jumping, skipping, side-stepping, and marching. There was a significant improvement of 2% for BMD at the trochanter in the exercise group [67], whilst participants in the control group experienced a parallel 2% decrease in BMD over the 12-month period. However, participants also performed exercises focused on improving muscular endurance, along with light-intensity aerobic activity, and as per other studies it remains difficult to ascertain the effect of the high-impact exercise alone.

Similar results have been obtained by Allison et al., who conducted a 12-month high-impact intervention in a cohort of 50 healthy adult men. The study involved three weekly sessions of unilateral vertical hops, before progressing to daily multidirectional single leg hops, following the first eleven weeks of the intervention. The interventional limb demonstrated significant improvement of +1.6% in femoral neck BMD compared to the control limb of participants. Interestingly BMD improved significantly within L4 vertebrae (+1.4%, *p* = 0.04), but not in L1–L3 (All *p* > 0.05) [73]. Such findings indicate that high impact exercises such as hopping may be most beneficial at distal sites (closer to the ground), which would be expected to experience the greatest GRFs.

Conversely, high-impact exercise alone, in the form of 50 jumps, completed six days a week, was not able to produce positive BMD change in a cohort of 124 post-menopausal women [28]. Korpelainen et al. also failed to demonstrate a significant positive influence of high-impact activity in a study of 160 post-menopausal women. In this cohort, the 30-month intervention was not associated with BMD changes in the exercise group, yet significant decreases at the femoral neck and trochanter of −1.1% and −1.6%, respectively, were observed in the control group [28].

There is currently a lack of adequately powered randomised control studies investigating the efficacy of strictly enforced high-impact activity for improving BMD, particularly in older osteoporotic or osteopenic adults. As can be seen in Table 1, sample sizes vary markedly in these interventions, with studies from Welsh et al. and Bassey et al. had particularly low statistical power. Importantly, these studies are unlikely to demonstrate significant effects of exercise. There is also no consensus regarding how high-impact interventions can be most effectively prescribed. Encouragingly however, trials to date have typically demonstrated that high-impact activity is associated with improvements or maintenance of BMD. Furthermore, a recent RCT demonstrated that high-impact exercise can improve bone health without any detrimental effects on knee cartilage composition in post-menopausal women with knee osteoarthritis, suggesting that this form of exercise does not increase the risk of musculoskeletal injury [71]. Future research should aim to move towards a consensus on high-impact exercise protocols, as well as developing clinical guidelines and public health messages to promote this form of exercise, which is widely accessible but has low participation rates in older adults.

### 3.4. Whole-Body Vibration Therapy

Whole-body vibration therapy (WBVT) is a form of passive activity that has demonstrated positive outcomes for a range of musculoskeletal disorders [83,84,85]. Whilst not regarded as a traditional mode of exercise, WBVT operates mechanistically like other exercise modalities, by inducing stress within the musculoskeletal system, causing subsequent adaptation. Little more is required from individuals than standing on a rapidly oscillating platform while maintaining balance and stability. WBVT can operate in a number of planes; small rotational oscillations around a central axis, up and down in the vertical plane, or side-to-side in the lateral plane. This movement generates forces that are subsequently transferred to the weight-bearing bones of the skeleton as per other modes of exercise [86]. 

Cross-sectional studies have demonstrated that occupational vibration exposure, such as that encountered whilst using power tools, may have negative effects on musculoskeletal health parameters [87], but WBVT has demonstrated varying efficacy in regard to changes in BMD. In a trial of 108 post-menopausal women (68 ± 4 years) comparing various WBVT modes and devices, it was demonstrated that vertical vibration produced no benefit to lumbar spine BMD, yet rotational WBVT produced a significant but small positive change in lumbar BMD (+1.1%) compared with controls [88]. Similarly, Verschueren and colleagues showed that thrice-weekly WBVT for six months in a group of post-menopausal women increased total hip BMD by 0.93% compared with controls [89]. Gusi et al. demonstrated positive changes of 4% in BMD at the femoral neck for WBVT compared to a walking exercise group [90], whilst Turner et al. amongst others have reported changes in bone turnover markers suggestive of osteogenic effects during the course of therapy [91,92]. However, it is important to recognise that the study from Gusi et al. recruited 28 participants, and as a result of low statistical power the 4% observed increase may be more attributable to a type 1 statistical error than WBVT itself.

Conversely, Leung et al. reported that a course of 18-month high-frequency WBVT in a randomised control trial (*n* = 710) did not significantly increase BMD in a population of 364 post-menopausal women (age 75 ± 7 years). There was, however, a significant decrease in the number of falls and fractures within the vibration therapy cohort compared to the control (*n* = 346) [83], indicating that vibration therapy may reduce the burden of osteoporosis, although not via increases in BMD. 

The variation within available systems, operating in vertical or rotational modes, and varying intervention lengths, along with sample sizes and delivery methods, likely explain the conflicting evidence for efficacy of WBVT in improving bone health. The differing protocols are briefly summarised in Table 2.

Moreover, whilst this passive activity has been associated with few adverse events, questions have been raised regarding its safety, particularly an increased potential for falls as a result of the oscillating activity [93]. These concerns are reasonable for older adults with osteoporosis and functional deficit, and may explain the relative scarcity of research in these populations. A recent systematic review discussed results from WBVT trials in osteopenic and osteoporotic older adults [94], with only one of five studies demonstrating an improvement in BMD [95]. Contrastingly, a study from Karakiriou et al., who conducted WBVT at a frequency of 35–40 Hz, substantially higher than many other studies, observed an improvement in lumbar spine BMD (+1.96% vs. controls), suggesting that higher frequency oscillation may be necessary for BMD gains in older adults. However, it should be noted that intra-group improvement was only +0.37% (*p* > 0.05) [95], indicating that WBVT may be more effective for BMD maintenance, rather than anabolism, in older adults with osteoporosis.

The efficacy of WBVT in reducing the burden of osteoporosis remains unclear given the relatively limited and conflicting robust interventional studies in older adults with osteoporosis. Moreover, trials to date that have demonstrated positive associations have generally been of lower quality. However, it is possible that WBVT may positively influence BMD in at-risk populations, particularly when performed at higher frequencies. High-quality trials are required to confirm the efficacy and safety of high-frequency WBVT in older adults with osteoporosis.

## 4. Conclusions

PA and exercise hold promise as primary or adjunctive therapies for prevention and treatment of osteoporosis. However, varying modalities may have differential effects on osteogenesis, and within each form of exercise, the significant heterogeneity in overall study quality currently limits inferences regarding their therapeutic efficacy.

Nevertheless, interventional studies have demonstrated improvements in bone health in both older men and women. Whilst the strength of associations and prospective changes in BMD might be regarded as modest, it is important to recognise that BMD is only one factor important in reducing fractures, a seemingly small 1–3% improvement in BMD may be sufficient to avoid a fracture. Importantly, these increases are similar to those seen with anti-resorptive drugs that reduce vertebral and non-vertebral fracture rates [96]. Although initial exercise uptake and maintenance are common concerns for clinicians, it should be noted that exercise compliance is not necessarily poorer than that of pharmacological regimes for osteoporosis [97]. 

Characteristics of bone other than BMD, such as geometry and morphology, are independently associated with fracture risk, with research indicating that these characteristics also respond to various forms of PA. Thus, reducing the burden of osteoporosis may be achieved via a number of pathways, with improvement in BMD representing only one of many important factors. In particular, exercise and PA that additionally result in improvements in muscle strength and increased balance and joint proprioception are likely to reduce the risk of falls and therefore indirectly lower fracture risk [98]. Exercise programs developed for fall prevention have been shown in meta-analysis to reduce falls by up to 39% in community-dwelling older adults [99]. Such interventions may offer an additional benefit for bone health, through promotion of increased weight-bearing PA and PRT, although a randomised control trial of the Otago and FaME falls prevention programs demonstrated no positive effect on BMD in older adults [100].

In conclusion, research demonstrates that free-living PA and exercise are associated with both cross-sectional and prospective significant but modest improvements in BMD and, at the very least, appear to exert homeostatic influences on BMD during ageing. Specifically, research appears to indicate that resistance training and weight-bearing activity may be most efficacious for maintaining and increasing BMD in older adults. A challenge in prescribing PA as a therapy for osteoporosis is supporting adoption and compliance to exercise protocols. It has been demonstrated that lack of time and access to transportation are the most commonly reported barriers to exercise participation in patients with osteoporosis [101]. Thus, clinicians and researchers should explore strategies to facilitate exercise participation in this population, such as the safety and efficacy of home-based impact exercise protocols. In support of this, future research should work towards developing methods of objectively monitoring participation in PA and exercise specifically targeting osteogenesis, particularly the measurement of GRFs in long-term interventions. Finally, the development of standardised guidelines for exercise and bone health in older adults and those with osteoporosis will make important contributions to clinical guidelines for osteoporosis management and public health messages to prevent fractures. 

## Figures and Tables

**Table 1:** Summary table highlighting differences in selected studies examining the effect of high-impact exercise on bone mineral density in randomised control trials.

	Population	Intervention	Length	Intervention Group BMD Change	Control Instructions	Control group BMD Change
Korpelainen et al. [28]	Elderly women (*n* = 160)	20 min daily unsupervised & 1 h intermittent supervision	30 months	↔Femoral neck	Daily PA	↓Femoral neck ↓Trochanter
Welsh et al. [67]	Men & women (*n* = 30)	Supervised exercises sessions 2–3/week	12 months	↑ Femoral neck	Daily PA	↓Femoral neck
Bassey et al. [82]	Postmenopausal women (*n* = 44)	50 ‘heel drops’ daily	12 months	↔ Lumbar spine ↔Femoral neck	Weekly exercise class	↔ Lumbar spine ↔Femoral neck
Allison et al. [73]	Older men (*n* = 50)	50 unilateral hops/day	12 months	↑ Femoral neck ↑ L4	Daily PA	↓Femoral neck

↑: Indicate Gain; ↔: Maintenance; ↓: Decrease in BMD.

**Table 2:** Summary of selected Whole Body Vibration Training studies in post-menopausal women.

	Population	Modality	Frequency	Length	Exclusions	Outcomes	BMD Change
Von Stengel et al. [88]	Postmenopausal women (*n* = 108)	Vertical: 35 Hz Rotational: 12.5 Hz	3x/week	12 months	Diseases or medication affecting bone	DXA BMD Isometric strength	↑Lumbar spine
Leung et al. [83]	Women ≥ 60 years (*n* = 710)	Vertical: 35 Hz	5x/week	18 months	Disease or medication affecting bone	Falls & fracture DXA BMD	↔Hip
Verschueren et al. [89]	Postmenopausal women (*n* = 70)	Vertical: 35–40 Hz	3x/week	6 months	Osteoporosis or Medication affecting bone	DXA BMD C-Telopeptide Osteocalcin	↑Hip
Gusi et al. [90]	Postmenopausal women (*n* = 28)	Lateral: 12.6 Hz	3x/week	8 months	Osteoporosis or medication affecting bone High Impact PA	DXA BMD	↑Femoral neck
Turner et al. [91]	Postmenopausal women (*n* = 46)	Vertical: 12 Hz	1x/week & 3x/week	8 weeks	WBVT contraindications Bone disease other than osteoporosis	Alkaline phosphatase N-telopeptide	Not reported

↑: Indicate Gain; ↔: Maintenance; ↓: Decrease in BMD.
